# The Epidemiology, Cost, and Occupational Context of Spinal Injuries Sustained While ‘Working for Income’ in NSW: A Record-Linkage Study

**DOI:** 10.3390/ijerph15102121

**Published:** 2018-09-27

**Authors:** Lisa N. Sharwood, Holger Mueller, Rebecca Q. Ivers, Bharat Vaikuntam, Tim Driscoll, James W. Middleton

**Affiliations:** 1 Sydney Medical School, University of Sydney, Camperdown, NSW 2050, Australia; bvai6198@uni.sydney.edu.au (B.V.); james.middleton@sydney.edu.au (J.W.M.); 2 John Walsh Centre for Rehabilitation Studies, Kolling Institute, St Leonards, NSW 2065, Australia; 3 Department Epidemiology and Preventive Medicine, Monash University, Melbourne, VIC 3004, Australia; 4 The George Institute for Global Health, Newtown, NSW 2042, Australia; hmoeller@georgeinstitute.org.au (H.M.); rivers@georgeinstitute.org.au (R.Q.I.); 5 School of Public Health, University of New South Wales, Kensington, NSW 2033, Australia; 6 School of Public Health, University of Sydney, Camperdown, NSW 2050, Australia; tim.driscoll@sydney.edu.au; 7 Agency for Clinical Innovation, Chatswood, NSW 2067, Australia

**Keywords:** workplace injuries, spinal trauma, record-linkage data

## Abstract

This study aimed to describe the epidemiological characteristics, the occupational context, and the cost of hospitalised work-related traumatic spinal injuries, across New South Wales, Australia. A record-linkage study of hospitalised cases of work-related spinal injury (ICD10-AM code U73.0 or workers compensation) was conducted. Study period 2013–2016. Eight hundred and twenty-four individuals sustained work-related spinal injuries; 86.2% of whom were males and had a mean age of 46.6 years. Falls led to 50% of the injuries; predominantly falls from building/structures, ladders or between levels. Falls occurred predominantly in the construction industry (78%). Transport crashes caused 31% of injuries and 24% in heavy vehicles. Half of all the transport injuries occurred ‘off road’. The external cause was coded as ‘non-specific work activity’ in 44.5% of cases; missing in 11.5%. Acute care bed days numbered at 13,302; total cost $19,500,000. High numbers of work-related spinal injuries occurred in the construction industry; particularly falling from a height. Off-road transport-related injuries were significant and likely unaddressed by ‘on-road’ prevention policies. Medical record documentation was insufficient in injury mechanism and context specificity. Workers in the construction industry or those using vehicles off-road were at high risk of spinal injury, suggesting inefficient systems approaches or ineffective prevention policies. Reducing the use of non-specific external cause codes in patients’ medical records would improve the measurement of policy effectiveness.

## 1. Introduction

Traumatic spinal injuries (TSI) can comprise column fractures, spinal cord injury or both. They are among the most severe injuries with potential long-term, physical, psychological, and social consequences. The leading causes of TSIs are widely reported as falls and motor vehicle crashes [1,2,3,4,5]; some of these occur in occupational settings, placing a significant financial burden on both employer and employee, the compensation system, and the health care system. Safe Work Australia calculated the cost of work-related falls to be at $6,640,000, in 2012–2013 [6,7]. The economic cost of work-related injury and illness, more broadly, has been estimated at ranging from 1.8% to 6.5%, of a country’s gross domestic product [8]. Safe Work Australia’s current “Work Health and Safety Strategy” [9] has set one of its three national targets as a 30% reduction in serious workplace injury compensation claims, by 2022.

The global epidemiology of TSI has recently been estimated in a systematic review [10]. The modelled overall global incidence was 10.5 cases per 100,000 persons; meaning around 768,000 new cases across the world per year. These authors reviewed 102 studies, reporting the TSI from work-related falls to be more common in low-to-middle-income countries, than in high-income countries. Road traffic crashes and falls were the most common mechanisms of injury in all income strata, however, low-to-middle-income countries had a higher proportion of cord injuries, only, as part of the injury profile in TSI. While comprehensive, this review was unable to report, more specifically, the industry context of the work-related TSIs. Numerous studies describing TSI epidemiology have been single hospital or trauma centre-based only and have not apportioned attributable risk to work-related incidents [2,3,11]. The authors recognised the paucity of the literature, globally, in examining TSI in population-based studies [10], particularly relative to the preponderance of studies considering solely cord injuries. If we are to consider only injuries to the spinal cord, and which are attributable to work globally, this was estimated at between 10–25%, in 2001 [12]. More recently, the Australian Institute for Health and Welfare (AIHW) reported the incidence and prevalence of traumatic spinal cord injuries across Australia [13]; “working for an income” was the second most common specified activity type at the time of injury, over the five-year period; accounting for 13% of all spinal injuries. A notable limitation of the AIHW data is that it is compiled using only data provided by consenting patients from the participating spinal cord injury units (SCIUs), in Australia, therefore likely to be an underestimate, based on an analysis of state-wide record-linked data, in New South Wales (NSW) [14]. Further, a vital missing component of these reports is the examination of the activity, at the time of injury.

The primary aim of this study was to describe the population-based epidemiology and occupational context of hospitalised traumatic spinal column and cord injuries, that occurred while ‘working for an income’ in NSW, the most populous state in Australia. The study does not include work-related ‘back’ injuries, for example, only musculoskeletal injuries without any spinal column or cord injuries. Traumatic spinal cord and column injuries are severe, and highly unlikely to avoid a period of hospitalisation; hence, the capacity of this study to accurately describe the health system burden of these injuries. A secondary aim was to describe the completeness of activity-coded data.

## 2. Methods

The study is set in the most populous Australian state—NSW, with approximately 7.5 million inhabitants, spread over 800,000 km^2^ in suburban, rural, and very remote areas [15]. The study time period covered from 1 June 2013–30 June 2016.

Study inclusion criteria were: patients aged ≥16 years, who had a recorded TSI as the reason for their index admission and an indication that this injury was related to work. Work-relatedness was defined by the International Classification of Diseases and Related Health Problems, 10th Version, Australian Modification (ICD10-AM) [16] code U73.0, or at least some of the costs covered by funding from workers compensation, in either the index admission or any subsequent episode of care within the acute care continuous period of stay.

Study exclusion criteria were: patients aged <16 years, and those for whom the first admission with a record of spinal injury was for rehabilitation.

Spinal injuries included all traumatic spinal cord injuries or spinal column injuries, defined on the basis of specific ICD-10-AM [16] codes (Appendix A). Probabilistic data linkage was undertaken by the Centre for Health Record Linkage (CHeReL), linking all patients where a TSI code was either a principal or additional diagnosis, for any separation within the Admitted Patient Data Collection (APDC). The APDC contains records for all patients admitted to all public hospitals across the state of NSW. Corresponding records were linked with the Emergency Department (ED) Data, NSW Mortality data, NSW Ambulance data collections, and the NSW District Network Return (DNR) activity-based funding data. The NSW APDC contains all inpatient records including patient demographics, comprehensive admission, transfer and discharge data, ICD-10-AM [16] diagnosis codes, Australian Refined-Diagnostic Related Group (AR-DRG) codes, procedure codes, separation mode (discharges, transfers and deaths), and financial information from all NSW public hospitals, private hospitals, and day procedure centres. The NSW DNR records the individual patient-level health service activity, enabling the estimation of the costs incurred by the health service providers, in a bottom-up approach. The first hospital episode for the patient, satisfying inclusion criteria conditions, as well as all other contiguous episodes of care, including nested/non-nested transfers, was recognised as the ‘index event’. The Socio-Economic Indexes for Areas (SEIFA); developed by the Australian Bureau of Statistics, to rank areas in the country according to the relative socio-economic disadvantage or advantage, was derived from the patients’ residential postcodes to describe the population relative to their education and occupation, within this index.

Activity and the place of injury were derived from the first record, or from subsequent records of the first continuous period of stay, if relevant information was missing in the first record. The sub-codes of the root U73.0 were used to identify the attributed industry (U73.00–U73.09). Type of TSI and body region affected was derived from any record within the first continuous period of stay. Length of stay was calculated for the first continuous period of stay. Costs were derived from the NSW DNR, which included the ED and the admitted costs.

Descriptive statistics were used to report the prevalence of various factors. Values were reported as the mean and the standard deviation (SD), in normally distributed continuous variables, or proportions; median and interquartile range (IQR) for non-normally distributed continuous variables. All statistical analyses were performed using Stata version 15.0 (Stata Corporation, College Station, TX, USA). Standardised reporting of the demographic and other variables, as recommended by De Vivo et al. [17], was followed where possible. The incidence of injury was based on the NSW Labour Force, aged 15 years and over [18],—note that the numerator data are based on persons 16 years and over—with 95% confidence intervals calculated, assuming a Poisson distribution [19].

This study was approved by the Cancer Institute NSW, Population and Health Services Research Ethics Committee: AU RED Reference: HREC/16/CIPHS/19, Cancer Institute NSW reference number: 2016/07/647.

## 3. Results

From this record-linked administrative dataset, 824 patients aged 16 years or over were identified as having sustained a TSI in NSW, while working for an income over the period June 2013–June 2016. Injury victims were predominantly male (86.2%), with a gender ratio of 6.22. Almost half of these injuries occurred as a result of a fall (49.2%), and more than half of these (55.1%) were falls from building structures, scaffolding or ladders (Table 1). Of the 254 (31%) transport-related spinal injuries, 50% occurred in an off-road setting. Fewer workplace injuries occurred in summer (20%), compared with other seasons, but the variation between seasons was not substantial (note that the proportion of ‘winter injuries’ includes one more month—June 2013—than the other seasons). Workplace-related spinal injuries represented almost 5% of all acute TSI, identified in this dataset.

Table 2 shows that the most common injury was a fracture and/or dislocation to the lumbosacral region (58%). Comorbid head, chest or abdominal injury was also sustained in 21% of incidents. Table 2 also quantifies the measure of resource required caring for these injuries. The mean length of the acute-care stay was more than two weeks (16.1 days). The total number of acute-care bed days, used by the 824 injured persons, was 13,302 at a total cost of $ 19,500,000 (95%CI $16 M–$23 M). The mean (SD) per patient cost for acute admission was $23,681.00 ($62,304.00); median (IQR) per patient cost was $7436.50 ($3381.50–$20,393.00).

For the 47 patients who had inpatient rehabilitation (130 hospital separations), the mean (SD) length of stay in rehabilitation was 40 days (44).

Table 3 further investigates the activity within each broad mechanism of injury category, permitting a greater understanding of the circumstances of injury, within certain activities. The most common type of fall injury was related to falling from a building or structure (24.4%), while the most common transport injury was sustained as an occupant of a heavy vehicle (23.6%). Over half the workplace TSIs caused by a mechanical force resulted from being struck by a projected or falling object (54.1%).

The external cause activity was coded as ‘other or unspecified’ in 44.5% of cases, and ‘missing’ in another 11.5% of cases (Table 1). Of the remaining cases where codes were specified to an industry, 78.8% of all falls occurred to persons in the construction industry, as did 48.3% of all mechanical force injuries. Transport-related injuries occurred predominantly in the transport and storage industry (51%) or agriculture/forestry industry (41.5%), and rarely in the construction industry (4%).

The annual rate of workplace-related spinal injuries was 7.2 (95% CI 6.8–7.7) per 100,000 persons, with the rate per 100,000 persons for males (11.6, 95% CI 10.7–12.5) being six times that of females (2.2, 95% CI 1.8–2.6).

## 4. Discussion

This study identified 824 persons during a three-year period who sustained TSIs while working for income. Almost half of these injuries resulted from a fall (49.2%); often from building structures, scaffolding or ladders (55.1%). Given that only 18% of TSI occurred in the 16–29 years age group, developmental vulnerability, and inexperience do not seem to be major contributing factors. The SEIFA index classifying education and occupational status shows a slightly higher proportion in the lowest three quintiles (67%), than in the higher two. The only comparator to this study analysed NSW hospitalisation and workers compensation data almost 20 years ago and considered all injury types, and not TSI alone [20].

Almost half of all work-related spinal injuries in our study were due to falls. Fall risk in Australian workplaces has been the focus of national attention over recent years. A Working at Heights Association survey [21], in 2014, found concerning failure rates of unsafe equipment installation, where 94% of fixed ladders were identified as potentially fatal. Despite numerous publications of codes of practice promoting workplace health and safety (WHS), and strong WHS laws, our results suggest that increased local surveillance of safety systems and stricter enforcement of relevant legislation may be required to reduce risks and, therefore, fall-related injuries.

Heavy transport vehicle crashes were the leading cause of transport-related spinal injuries in this study. A heavy vehicle driving crash risk is known to be reduced by the consumption of caffeinated substances [22], however, increased with night shift driving, insufficient breaks, and lack of vehicle safety devices [23]. Industry safety for heavy vehicle drivers has a long chain of responsibility that involves general practitioners in driver licensing, logistics managers, employers of various sizes, loading managers, goods consigners, and many others. Where multiple parties may be responsible at different stages in risk profiles, it is clear that all parties must work seamlessly together to reduce overall risk.

The reliability of the external cause codes in ICD10-AM [16] has been previously proven to be questionable, with high levels of missing activity codes, and ‘non-specific’ coding to describe events surrounding injury cause [24,25]. Analysis of a nationally representative sample of injury admissions across Australia, in 2002, revealed an underestimation of around 32%, in hospital coding, of the true burden of occupational injury [26]. The sub-codes of the root U73.0, used to identify the attributed industry (U73.00–U73.09), within which the activity was being undertaken at the time of injury; was missing or coded as ‘other’ or unspecified, in more than half of all cases (56%). Lack of coding specificity clearly hampers the provision of information to injury prevention activities and policy and likely leads to underestimation of actual events. The importance of improving the quality of clinical documentation, and particularly the level of specificity surrounding the injury events has been previously highlighted by Soo et al. [25]. These authors suggested that this perhaps indicated a relatively lower perceived importance of activity information, compared to injury mechanism codes, which have been more completely coded [25].

A particular strength of this study is its comprehensive profile of work-related TSIs, drawn from a population-based record linkage; not previously offered in Australia. Despite the undoubted under-reporting due to medical coding issues, these data aptly inform the current policy targets from the Australian Work Health and Safety Strategy 2012–2022 [9], to achieve a “reduction in the incidence rate of claims resulting in one or more weeks off work of at least 30%” for workplace injuries. Baseline data for this policy included serious non-fatal workers’ compensation claims, reportedly around 11.5 claims per 1000 employees, over the 2009–2010 to 2011–2012 period. The objective of 30% reduction is acknowledged as ‘ambitious’ but thought to be attainable, if ‘concerted and sustainable effort is made to target the most common causes of injury in those industry sectors that experience both high numbers and high rates of injury’ [27]. This study did not consider TSIs related to other workplace injuries, however, this injured population certainly would be included within a ‘30% reduction’ target. A replicated follow-up record linkage, in 2022, would ideally quantify the measure of any reduction achieved.

Limitations of this study included the unavailability of various important variables about the injured worker, such as ethnicity, level of education and experience, the employment situation (e.g., whether permanent/part-time/casual), and specific occupation. Indigenous status was not identified within the APDC collection; the population of NSW has 2.9% of indigenous people. These variables may influence the risk profile within particular industries or worker groups. The source data coding issues have been discussed earlier. Finally, the APDC does not include hospitalisation data for patients who were admitted to private hospitals across NSW. The degree of under-representation that this presents is uncertain, however, such severe injuries are much more likely to be treated within the public hospital system.

## 5. Conclusions

Work-related traumatic spinal injuries create a significant burden of cost and disability for the Australian workforce but are preventable, and also fall under a current focus of the Safe Work Australia policy to reduce serious injury compensation claims, by 30%, by 2022. This study demonstrates that the construction industry is still experiencing a high burden of work-related spinal trauma, particularly related to falling from heights, with a need for more effective policies, risk management strategies, and countermeasures for prevention. Transport injury prevention efforts equally need to address heavy vehicle occupant risk. Further research is required to better understand the factors contributing to traumatic spinal injuries in the workplace, including worker risk profiles, job design, work environment, culture, and leadership. Finally, the design and tailoring of industry-specific injury prevention strategies would be greatly assisted by improving the quality of medical record documentation, including, specifically, a reduction in the use of non-specific external cause codes. A more accurate identification of the activities and sectors which give rise to workplace spinal trauma, has policy relevance, as injury circumstances inform public health initiatives to target injury prevention.

## Figures and Tables

**Table 1:** Characteristics of the patient population and context of injury occurrence (*n* = 824).

Characteristics	*n* (%)
Sex	
- male	710 (86.2)
- female	114 (13.8)
Age category years	
- 16–29	145 (17.6)
- 30–44	233 (28.3)
- 45–59	280 (33.9)
- 60–74	144 (17.5)
- 75+	22 (2.7)
Season of injury	
- Summer	165 (20.0)
- Autumn	205 (24.9)
- Winter	230 (27.9)
- Spring	224 (27.2)
Mechanism of injury (more detail Table 3)	
- Falls (W00–W19)	405 (49.2)
- Transport (V01–V99)	254 (30.8)
o Traffic	127 (50.0)
o Non-traffic (off-road)	127 (50.0)
- Mechanical forces (W20–W64)	135 (16.4)
- Other and unspecified (X58–X59)	30 (3.6)
Place of injury occurrence	
- Industrial and construction area	161 (19.5)
- Street and highway	124 (15.0)
- Farm	84 (10.2)
- Trade and service area	57 (6.9)
- Home	37 (4.5)
- Sports and athletics area	27 (3.3)
- Other specified	49 (5.9)
- Unspecified	285 (34.6)
Industry (external cause—within activity code)	
- Construction	163 (19.8)
- Agriculture, forestry, and fishing	104 (12.6)
- Transport and storage	82 (9.9)
- Manufacturing	13 (1.6)
- Other specified work for income	183 (22.2)
- Unspecified	184 (22.3)
- Missing	95 (11.5)
SEIFA Index (Education/Occupation: quintiles) *	
- First quintile ^#^	138 (17.3)
- Second quintile	193 (24.1)
- Third quintile	202 (25.3)
- Fourth quintile	151 (18.9)
- Fifth quintile ^##^	116 (14.5)
- Missing	24 (2.9)

* derived from patient’s residential postcode ^#^ lowest education and occupation status ^##^ highest education and occupation status.

**Table 2:** Injury detail and consequent hospitalisation.

Injuries Sustained	Number	%
Spinal cord injury	62	7.5
Cervical level fracture/dislocation	183	22.2
Thoracic level fracture/dislocation	295	35.8
Lumbosacral level fracture/dislocation	480	58.3
Co-morbid traumatic brain injury	65	7.9
Co-morbid severe chest injury	93	11.3
Co-morbid severe abdominal injury	33	4
Subsequent rehabilitation admission = yes	47	5.7
Length of stay acute care days (mean (SD))	16.1	(39.7)
Length of stay acute care days (median (IQR))	4.9	(1.7–11.5)
Length of stay rehabilitation days (mean (SD))	40	44
Length of stay rehabilitation days (median (IQR))	24.3	(13.9–45.9)

**Table 3:** A detailed description of injury in the context of activity.

Specific Activity Leading to Injury	*n* (%)
Falls	
- From building/structure	99 (24.4)
- On and from ladder	91 (22.5)
- Other from one level to another	77 (19.0)
- On and from scaffolding	33 (8.1)
- On and from stairs	27 (6.7)
- Same level	24 (5.9)
- Other same level	22 (5.4)
- Other & unspecified	21 (5.2)
- From tree	11 (2.7)
Total falls	405 (100)
Transport accidents	
- Occupant of heavy transport vehicle (V60–V69)	60 (23.6)
- Car occupant (V40–49)	48 (18.9)
- Animal-rider (V80)	37 (14.6)
- Motorcycle (V20–V29)	21 (8.3)
- Occupant of special all-terrain vehicle (V86)	21 (8.3)
- Pedestrian (V01–V09)	12 (4.7)
- Occupant of pick-up truck (V50–V59)	11 (4.3)
- Other and unspecified (V98–V99)	11 (4.3)
- Occupant-vehicle mainly used in agriculture (V84)	9 (3.5)
- Other	9 (3.5)
- Pedal cycle (V10–V19)	8 (3.1)
- Occupant-vehicle used on industrial premises (V83)	7 (2.8)
Total transport accidents	254 (100)
Mechanical forces	
- Struck by thrown, projected or falling object (W20)	73 (54.1)
- Bitten or struck by other mammals (W55)	21 (15.6)
- Contact with other and unspecified machinery (W31)	17 (12.6)
- Striking against or struck by other objects (W22)	13 (9.6)
- Other specified	11 (8.1)
Total mechanical forces	135 (100)

## Appendix A

ICD10-AM Codes provided to the Centre for Health Record Linkage, in order to identify persons aged 16 years or over, at admission, who were admitted after sustaining a traumatic spinal injury: S12, S12.0, S12.1, S12.2, S12.21, S12.22, S12.23, S12.24, S12.25, S12.7, S12.8, S12.9, S13.1, S13.10, S13.11, S13.12, S13.13, S13.14, S13.15, S13.16, S13.17, S13.18, S13.2, S13.3, S14.0, S14.10, S14.11, S14.12, S14.13, S14.70, S14.71, S14.72, S14.73, S14.74, S14.75, S14.76, S14.77, S14.78, S22.0, S22.00, S22.01, S22.02, S22.03, S22.04, S22.05, S22.06, S22.1, S24.0, S24.1, S24.10, S24.11, S24.12, S24.7, S24.70, S24.71, S24.72, S24.73, S24.74, S24.75, S24.76, S24.77, S32, S32.0, S32.00, S32.01, S32.02, S32.03, S32.04, S32.05, S34.0, S34.1, S34.3, S34.70, S34.71, S34.72, S34.73, S34.74, S34.75, S34.76, T06.0, T06.1, T09.3.
